# Teaching at the intersection of science and society: An activity on healthcare disparities

**DOI:** 10.1093/biomethods/bpad041

**Published:** 2024-01-05

**Authors:** Paula E Adams, Enya Granados, Abby E Beatty, Cissy J Ballen

**Affiliations:** Department of Biological Sciences, Auburn University, Auburn, AL, USA; Department of Curriculum and Teaching, Auburn University, Auburn, AL, USA; Department of Biological Sciences, Auburn University, Auburn, AL, USA; Department of Biology, St. Mary's College of Maryland, St. Mary's City, MD, USA; Department of Biological Sciences, Auburn University, Auburn, AL, USA

**Keywords:** ideological awareness, inclusive teaching, healthcare disparities

## Abstract

Understanding the relationship between science and society is an objective of science education and is included as a core competency in the AAAS *Vision and Change* guidelines for biology education. However, traditional undergraduate biology instruction emphasizes scientific practice and generally avoids potentially controversial issues at the intersection of biology and society. By including these topics in biology coursework, instructors can challenge damaging ideologies and systemic inequalities that have influenced science, such as biological essentialism and health disparities. Specifically, an *ideologically aware curriculum* highlights how ideologies and paradigms shape our biological knowledge base and the application of that knowledge. Ideologically aware lessons emphasize the relationship between science and society with an aim to create more transparent, scientifically accurate, and inclusive postsecondary biology classrooms. Here we expand upon our ideologically aware curriculum with a new activity that challenges undergraduate biology students to consider the impacts of healthcare disparities. This lesson allows instructors to directly address systemic inequalities and allows students to connect biomedical sciences to real-world issues. Implementing an ideologically aware curriculum enables students to challenge prevailing worldviews and better address societal problems that lead to exclusion and oppression.

## Introduction

Integrating science and society in science education is an important goal for many organizations [1–3]. In the USA, the “Vision and Change” report laid out the core competencies for undergraduate biology education, which included understanding the relationship between science and society [2]. However, in a recent assessment of the implementation of the Vision and Change recommendations, authors found that overall, societal topics are rarely included in undergraduate biology courses [4]. When they are included, they are most likely found in smaller advanced courses rather than introductory courses, which educate a larger body of students with diverse educational trajectories. Introducing students to biology as “value-free” and disconnected from society in their first year may leave lasting impressions of its actual reach and influence [5, 6]. The lack of inclusion of science and society in undergraduate biology education communicates to students that biology and society are unrelated entities and we should avoid discussions of controversial issues that sit at their intersection. To bridge this gap in our teaching, we have developed course materials intended for introductory biology courses that enable instructors to implement socially relevant activities in the classroom [7–10].

### Promoting ideological awareness in introductory biology

One way to integrate societal topics into biology content is to clarify how personal views and beliefs have historically and currently affected the way science is conducted [5, 6]. Ideological awareness is an undergraduate biology curriculum resource for supplementing traditional biology course content with active learning lessons focused on addressing and challenging structural inequities in biological and biomedical sciences [7, 8]. Ideological awareness is grounded in culturally relevant pedagogy developed by Gloria Ladson-Billings [11–14]. Culturally relevant pedagogy provides biology students with relevant social context to address complex issues they will encounter in the society as medical professionals, research scientists, and cognizant citizens. Ideological awareness aligns with one of the central pillars of culturally relevant pedagogy: critical or sociopolitical consciousness [8]. Critical or sociopolitical consciousness allows students to “critique the cultural norms, values, mores, and institutions that produce and maintain social inequities” [13, 15]. Teaching through ideological awareness reveals the underlying values, biases, assumptions, and stereotypes that ultimately “inform approaches to and outcomes of science” [8].

### What are the impacts of the ideological awareness curriculum on students?

The ideological awareness curriculum has been implemented in a variety of settings (i.e. 2-year, 4-year, and R1 research-intensive schools). After a series of lessons ranging from unethical experimentations and its relationship to current research ethics, the use of science to justify societal suppression of nonconforming identities, and the lack of representation in science, technology, engineering, and mathematics (STEM) , Beatty *et al*. [7] found that students reported preferring these materials compared to traditional course content. Additionally, between 43 and 50% of the students were not familiar with the topics before the lesson was taught. Adams *et al*. [9] found that when asked to build a concept map of the biology course material, with explicit instructions to connect the material to aspects of society, students who did not receive an ideologically aware curriculum were unable to make societal connections (median = 0 societal topics included). Students in the ideologically aware section included more societal content and covered a greater variety of topics in their end-of-semester concept maps, while including the same amount of biological content [9]. Collectively, students were not previously familiar with these topics and did not make connections between science and society on their own. However, after instruction on these topics, they were capable of making the connections between science and society and reported preferring this material over the traditional biology textbook. Finally, we found that the inclusion of ideologically aware material did not come at the expense of traditional biology content coverage, as measured by the grade comparisons and the number of biology concepts students could identify at the end of the semester [7, 9].

### Instructors value ideological awareness, but express hesitancies

Our previous work underscored the importance of the instructor’s role in forging the connection between science and society for their students, as students struggle to make these connections without this explicit instruction [9]. Despite the documented benefits that we described above, why do we observe such low rates of inclusion of these materials in undergraduate biology courses? [4] A national survey of 128 biology instructors concluded that the main goal of science education was “understanding the world,” and instructors agreed that students should receive instruction about “the biases, stereotypes, and assumptions that shape contemporary and historical science” before they graduate [16]. Instructors reported that teaching ideological awareness is important because of increased student engagement and belonging in the class, its tendency to address misconceptions of science, and by providing students with connections between science and society. Despite their enthusiasm for the material, instructors were hesitant to include ideological awareness in their biology curricula [16]. Instructors may not include these topics because they perceived it took time away from coverage of existing core content, a lack of familiarity with the materials, and over concern that students are not prepared or mature enough for these discussions. When prompted to describe the worst-case scenario, instructors expressed fear over poor implementation, inadvertently alienating students or making them uncomfortable, and potential pushback from authorities (e.g. administrators, other faculty, outside forces) and termination. This reluctance may explain, in part, the scarcity of instructors who implement these materials in their courses. However, our previous work addresses some of these concerns, such as provisioning instructors with vetted course materials [current manuscript; 10], documentation of student preference for these materials [7], and positive student outcomes after teaching this material [9].

## Ideological awareness implementation

We have developed ideological awareness activities on a variety of topics that are tailored for undergraduate biology classrooms (Table 1). These activities are available in a regularly updated GitHub repository and include an instructor activity explanation, student worksheet, and lesson PowerPoint (Ideological Awareness activities available here: https://tinyurl.com/IdeologicalAwareness). Activities range from reading and discussing scientific articles and case studies to interacting with publicly available data [10]. Each instructor worksheet contains an activity description, suggested courses for implementation, learning goals and objectives, references, and adaptation suggestions for alternate implementations. All of these lessons have an active learning component that allows students to interact with and discuss these topics [17, 18].

**Table 1.** Summary of activities developed to promote ideological awareness in undergraduate biology.

Activity topic	Description
Environmental Injustice	Students discuss the basic principles of pollution, exposure to chemicals, and air pollution. Students use publicly available data to look at real-world examples of the disproportional implications of environmental degradation on oppressed populations
Integration of Evolution and Religion	Students will receive instruction on cultural competency and evolution, as well as examples from religious leaders and evolutionary biologists of faith. Students will then discuss the intersection of science and religion specifically relating to evolution
Tissue Ownership and Biological Ethics	Students will learn about the legality of tissue ownership in the context of HeLa cells collected from Henrietta Lacks. Through a discussion of research ethics, students will work in groups to build arguments about compensation for tissue donations that lead to financial or societal gains in science
Gender Identity and Sexuality	Students will read scientific articles and/or chapters related to organismal sex and sex determination. They will make connections to prior knowledge, learn appropriate terminology (for sex and gender concepts), and discuss the implications for both science and society
Representation in STEM	Students will read the summary of a research article about intersectional representation in introductory science textbooks [19]. Students will then scan their own textbooks for depictions of scientists to generate an authentic dataset and address a research question about representation in biology curricular materials [10]
Healthcare Disparities	Students will learn about the sources of healthcare disparities among people with historically excluded identities. Students will be assigned into groups to read a scientific article and build concept maps
Designer Babies	Students will learn about the new gene-editing technologies and CRISPR/Cas9. They will then explore the ethics of gene editing through the use of case studies of hypothetical pre-natal gene editing cases. They will discuss the ethical implications of gene editing and the societal implications

Below we provide readers with an extended example of one activity focused on healthcare disparities among people with excluded identities. First, we will offer an overview of the topic and follow with relevant information about the activity to guide readers who wish to implement this or a similar topic in their classroom. We provide an instructor worksheet (Supplementary Materials S2), student worksheet (Supplementary Materials S3), and slide deck for the introductory lecture and discussion (Supplementary Materials S4).

## Extended activity example: healthcare disparities among people with systemically excluded identities

Healthcare disparities are “a particular health difference that is closely linked with social, economic, and/or environmental disadvantage” [20]. Healthcare disparities disproportionately affect “groups of people who have systematically experienced greater obstacles to health based on their racial or ethnic group; religion; socioeconomic status; gender; age; mental health; cognitive, sensory, or physical disability; sexual orientation or gender identity; geographic location; or other characteristics historically linked to discrimination or exclusion” [20]. This topic has clear connections to ideological awareness, as many of these disparities are rooted in structural inequalities (e.g. access to affordable healthcare), discrimination (e.g. bigoted laws targeting transgender individuals), and values or beliefs (e.g. impacts on women’s ability to make reproductive decisions). This topic also has strong connections to biology and the future careers of many students who are taking biology, particularly those who wish to become medical professionals. In this activity, students read primary literature and constructed a concept map that required them to make explicit connections between the biology content and the societal topic. Finally, students are challenged to articulate the sources and impacts of healthcare disparities, as well as actionable strategies to reduce them.

### Learning goals

- Students will have a deeper understanding of the healthcare field by learning about the sources, effects, and solutions for healthcare disparities in marginalized communities.
- Students will know how to synthesize and connect concepts related to the sources, effects, and solutions of healthcare disparities from primary research.

### Learning objectives

Students will be able to:

- engage in critical thinking about implicit biases and healthcare inequalities;
- identify sources of healthcare disparities among minoritized individuals;
- identify strategies to reduce healthcare inequalities;
- compare central themes from various research topics;
- synthesize concept maps; and
- analyze disproportionate effects of healthcare disparities on intersecting identities.

### Intended audience

The activity was designed for and has been implemented in introductory biology courses for majors and non-majors. However, it could also be incorporated into courses beyond first-year biology sequences. For example, it would also be appropriate in upper-level coursework such as genetics, cell biology, health sciences, or any biomedical course. The slide deck engages students in a discussion of healthcare disparities and systemic inequities that affect marginalized communities (Supplementary Materials S4). Students will then read a scientific article on a focal healthcare disparity topic. Students are not required to have any disciplinary biology knowledge prior to completing this lesson but should be able to read and interpret scientific articles with guidance. This activity assumes students to possess prior knowledge of concept mapping. If students are not familiar with concept mapping, additional in-class and/or out-of-class prep may be necessary. Instructors should familiarize themselves with the scientific articles that students will be reading as well as information about healthcare disparities from the US Department of Health and Human Services, Office of Disease Prevention and Health Promotion reports *Healthy People 2020* & *Healthy People 2030* [20, 21].

### Lesson plan

The required in-class time for this activity is ∼75 min, and it can be completed in one or two class periods depending on the length of the class period and variations in implementation (see Table 2 for a timeline of course activities in ∼75-min class). In preparation for class, students will read an assigned article for homework (∼1 h). At the beginning of class, the instructor delivers a 15-min introductory lecture to provide context on the assigned articles and kickstart student discussions (Supplementary Materials S4). After the lecture, students will work collaboratively in their assigned scientific article groups to build their concept maps for the article (∼20 min; each student completes their own concept map which can be collected as an assessment), followed by building a full-class concept map covering all the healthcare disparity articles (∼20 min). The instructor should then wrap up the discussion and provide any closing remarks on the central themes of healthcare disparities across the scientific articles (>5 min). Students will then complete the assessment in class (∼15 min). The final whole-class concept map will allow students to become familiar with all the articles used in the activity and to build connections between the different factors contributing to healthcare disparities. The implementation of this activity will depend on the length of the class period. In a 50-min class period, the activity could be split over two class periods: in the first, the instructor delivers the introductory lecture and in the second, concept maps are built individually and as a whole class, with students reading the assigned scientific articles between these two class periods (see alternate timeline: Supplementary Table S1).

**Table 2.** Timeline of course activities in a 75-min class period.

Activity	Description	Est Time	Details
**Student preparation for class**			
Students read selected articles	In groups, students are assigned an article to read	<2 h	
Concept map introduction	Students will watch a video and read instructions on concept mapping		
**Class session components**			
Introductory lecture	The instructor reviews healthcare inequalities and connects them to prior knowledge throughout. The instructor introduces concept mapping (if new for this class)	>15 min	*PowerPoint included*
Collaborative work: concept mapping	Students break into groups to discuss their assigned scientific article and build their individual concept maps	>20 min	*Electronically or with paper/pencil*
Full-class concept-map	Dissolve groups; The class will work together to discuss their scientific articles and build a concept map combining ALL of the scientific articles	>20 min	*Electronically or with paper/pencil*
Discussion and wrap up	End the class with a discussion of the central themes/ideas that surround the idea of healthcare inequalities	>5 min	
Assessment	Students complete the post-activity assessment	∼15 min	*Included in student hand-out*

### Student preparation for class

Before class, students will be assigned to read one article within one of three focal topics: (i) socioeconomic disparities [22]; (ii) racial disparities [23–26]; or (iii) LGBTQ+ disparities [27–30]. While reading students are asked to reflection on the questions:

- What are the sources/causes of the healthcare disparities?
- What are the effects/impacts of the healthcare disparities?
- What are the strategies to reduce healthcare disparities?

**Figure 1.**
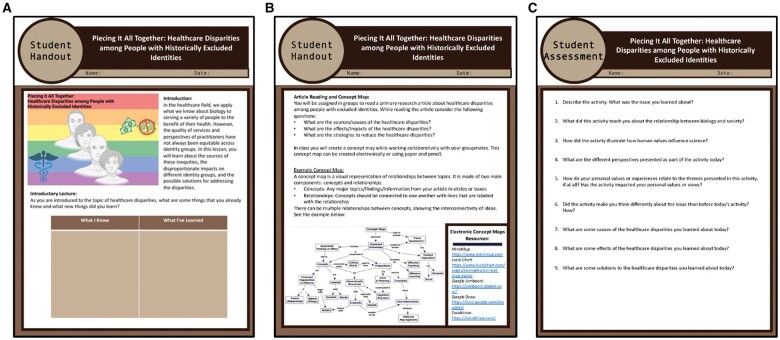
Students will receive a hand-out guiding them through the activity (Supplementary Materials S3). During the introductory lecture, they will be prompted to fill out the chart to reflect on what they know and what they have learned (**A**). Students also receive instructions on building concept maps (**B**). At the end of class, students will complete an assessment covering the topic of healthcare disparities (**C**).

They will then review introductory materials for concept mapping (Fig. 1B; Supplementary Materials S3).

### Introductory lecture

The introductory lecture sets the stage for the forthcoming activity and discussion. First, the instructor will define healthcare disparities and place the topic in the context of their course. They will describe how it is relevant to the careers of the students in class (many of whom may be interested in entering the medical field). The lecture then covers specific examples, such as the percentage of nonelderly adults who did not see a doctor in the past year, rates of uninsured individuals, maternal mortality, and enrollment in clinical trials by race/ethnicity. The instructor also discusses the barriers to healthcare access experienced by transgender Americans, ranging from intentional discrimination to insurance refusals.

During the lecture, students will reflect on the prompt “As you are introduced to the topic of healthcare disparities, what are some things that you already knew and what new things did you learn?” They will fill in a chart for “What I Know” and “What I’ve Learned” (Fig. 1A).

The instructor will then introduce concept mapping to the students using the provided instructor resources (Supplementary Materials S2).

### Collaborative work: concept mapping

After the introductory lecture, each student will build their concept maps for their assigned article while discussing the article with their group members. They are instructed to work collaboratively with and discuss the concepts or relationships between concepts with the group. We generally give students the option to build the concept map with pen and paper or electronically. The individual concept maps can be taken up at the end of the class and assessed for participation.

### Full-class concept mapping and wrap up

The instructor will then lead the classroom in the construction of a full-class concept map. The concept map can be drawn on a whiteboard or built with electronic concept-map building software and projected to a screen depending on the resources available in the classroom. Starting with the central theme of “healthcare disparities,” the instructor will invite students to share information about their assigned articles and connect their ideas to a larger class concept map. The instructor can prompt the students to share information with questions such as “what are the sources/causes of healthcare disparities?,” “what are the effects/impacts of those sources on the affected communities?,” and “what strategies can we use to reduce the causes and impacts of healthcare disparities?” Each group will share information directly from their assigned article including: (i) new concepts to add to the class’s model from their article/concept map; (ii) new relationships between concepts from their group’s article or concept maps; and (iii) adding new concepts or connection between existing concepts on the class concept map. The final concept map should provide a summary of the causes/sources of healthcare disparities, the impacts and effects of healthcare disparities on specific groups of people, and strategies for addressing the underlying causes of healthcare disparities. The instructor should end the class period with a discussion of the central themes and ideas surrounding healthcare disparities. If time allows, open the class to discussion or to share reflections on what students have learned throughout this activity.

### Assessment

The assessment can be completed at the end of class or as a post-class assignment. Ten short answer questions assess student knowledge of healthcare disparities and challenge students to connect biology content with societal topics (Fig. 1C).

1. Describe the activity. What was the issue you learned about?
2. What did this activity teach you about the relationship between biology and society?
3. How did the activity illustrate how human values influence science?
4. What are the different perspectives presented as part of the activity today?
5. How do your personal values or experiences relate to the themes presented in this activity, if at all?
6. Has the activity impacted your personal values or views?
7. Did the activity make you think differently about the issue than before today’s activity? How?
8. What are some causes of the healthcare disparities you learned about today?
9. What are some effects of the healthcare disparities you learned about today?
10. What are some solutions to the healthcare disparities you learned about today?

### Extensions/alternate assessments

There are several ways to modify the activity to suit the needs of individual courses (Supplementary Materials S2). For example, one way to extend the activity would be for students to submit a proposal outlining a plan of action for a solution to a particular healthcare disparity supported by the literature reviewed in class. If instructors are interested in additional forms of assessment, they can require students or groups of students to submit their concept maps for a grade (recommendations for concept map assessment can be found in Supplementary Materials S2). Finally, we recommend the following additional reflection or discussion questions that could be posed to students during class time:

- What three main themes did all of the research topics on healthcare disparities have in common?
- Describe the relationship between the cause, effect, and solution for a particular health disparity you learned about today.
- Describe a solution for a specific healthcare disparity you learned about today and discuss how it will impact the cause and effect of that healthcare disparity.
- Describe the intersectional (considering multiple aspects of one’s identity) effects of a particular healthcare disparity.

## Conclusions

Teaching ideological awareness in biology such as healthcare disparities is important for nonmajor and biology major student populations. This activity challenges students to consider structural systems of oppression that disadvantage people on the basis of their identities and circumstances, leading to inequalities in healthcare that can have dire consequences. We believe that the timing of discussions that can potentially transform how students view their place in education is critical and essential to deliver at the beginning of their science career (rather than in more advanced courses). Incorporating ideologically aware content into introductory coursework meets this need and invites students to question, challenge, and engage with curricular materials that sit at the intersection of biology and society.

## Supplementary Material

bpad041_Supplementary_Data

## Data Availability

No new data were generated or analysed in support of this research. Activity materials referenced in this article are available in the Supplementary materials and at https://tinyurl.com/IdeologicalAwareness.
