# Supplementary material for: Teaching at the intersection of science and society: An activity on healthcare disparities
Source: Biol Methods Protoc. 2024 Jan 5;9(1):bpad041. doi: 10.1093/biomethods/bpad041 (PMC10833140; doi:10.1093/biomethods/bpad041)
Supplement: bpad041_Supplementary_Data [file bpad041_supplementary_data.zip › S3_DispHealthcare_StudentHandout.pdf]

Student Handout

Piecing It All Together: Healthcare Disparities among People with Historically Excluded Identities

Name: Date:

Pre-course work: Become Familiar with Concept Mapping!

Please look through all of the links provided below in order to familiarize yourself with concept maps and how to properly produce this particular map.

1. Visit this webpage and watch the tutorial on completing concept maps: https://www.youtube.com/watch?v=8XGQGhli0I0
2. Visit the following links for more detailed written directions on concept mapping, as well as a guide on an alternative program "**cMAP**". https://www.evidencebasedteaching.org.au/concept-mapping-complete-guide/
3. Choose a platform to create your map. The above YouTube video uses a free web-based platform "**Lucid**". You are welcome to do that. Additionally, you may draw it by hand, or use one of the other following programs:
ClickCharts Diagram & Flowchart Software and **Gliffy** are web-based products that include built in templates to help you get started.
Visual Understanding Environment is a free open source software that you can download to your personal computer.
cMAP from the Florida Institute for Human and Machine Cognition (IHMC).
Microsoft Office products have many templates for displaying relationships and processes, The SmartArt feature is built into the Insert tab within Microsoft Word and PowerPoint that you can use to create your concept map
4. Check out this paper detailing the importance and usefulness of concept mapping as a learning tool. The author provides advice on using concept maps in regards to structure, feedback, exam alignment, and learning styles and study habits.
Reference: Weimer, M. (2015, January 20). Keeping students on board with concept maps. Faculty Focus. Retrieved from: http://www.facultyfocus.com/articles/instructional-design/keepingstudents-board-concept-maps/

Essential Reminders
Tips and tricks:
1. Use arrows to explain the connections between your key terms
2. Don't forget to show the interconnectivity of topics. Remember to show how concepts relate to each other. This is not a flow chart and should not be entirely linear. If you need to, look up additional information on the differences between flow charts and concept maps.
3. Touch on as many topics as possible- including specifics. Start with your main topics (genetics, anatomy, physiology, ecology, evolution, cells, the nature of science, etc.) and continue to build on those main topics to include more detailed explanations. For example, if you were concept mapping genetics, you may want to detail trait dominance, inheritance, Mendel, Punnett squares, pea plants, etc.). The connect inheritance to pea plants with the connection term explaining that pea plants were "experimented on" to learn about inheritance.
4. We want to know what you know, not what google knows. Each one of you will turn in a vastly different concept map, and that is okay! Do not panic about catching every little detail, just be a thorough as possible. This is open-note in the sense that you may use your notes , each other, and your textbook. Do not use the internet. This assessment is made to help you draw connections, and internet searches will not help you do that. We are very familiar with the results from such google searches, and we expect you to go beyond what can be found through such a search.
6. Be creative! Make jumps… don't get stuck on "my book doesn't say this". You will need to take the knowledge gained throughout the semester to draw new connections with the world around you. Those connections won't always be found in your text, and we do not expect citations. You will need to think critically, and outside the scope of information given to you explicitly in order to complete this assignment.

Student Handout

Piecing It All Together: Healthcare Disparities among People with Historically Excluded Identities

Name: Date:

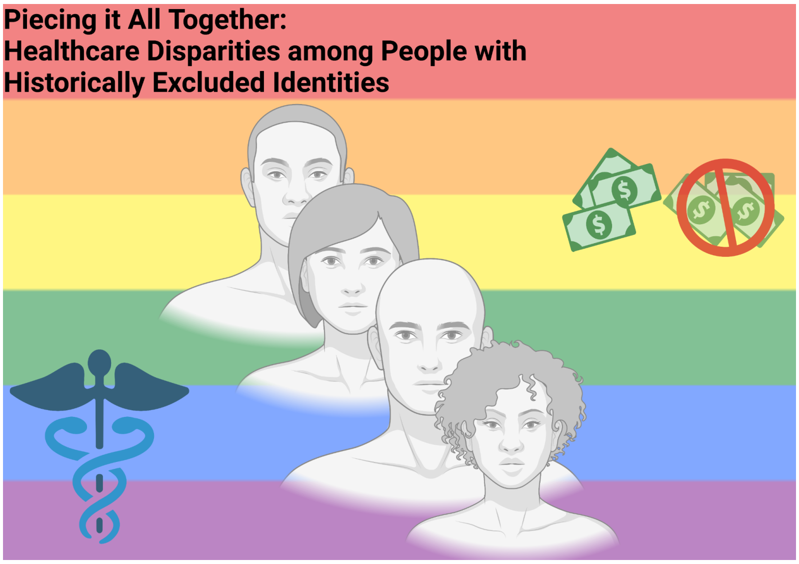

Introduction:

In the healthcare field, we apply what we know about biology to serving a variety of people to the benefit of their health. However, the quality of services and perspectives of practitioners have not always been equitable across identity groups. In this lesson, you will learn about the sources of these inequities, the disproportionate impacts on different identity groups, and the possible solutions for addressing the disparities.

Introductory Lecture:

As you are introduced to the topic of healthcare disparities, what are some things that you already knew and what new things did you learn?

What I Know	What I've Learned

Student Handout

Piecing It All Together: Healthcare Disparities among People with Historically Excluded Identities

Name: Date:

Article Reading and Concept Map:

You will be assigned in groups to read a primary research article about healthcare disparities among people with excluded identities. While reading the article consider the following questions:

- What are the sources/causes of the healthcare disparities?
- What are the effects/impacts of the healthcare disparities?
- What are the strategies to reduce the healthcare disparities?

In class you will create a concept may while working collaboratively with your groupmates. This concept map can be created electronically or using paper and pencil.

<u>Example Concept Map:</u>

A concept map is a visual representation of relationships between topics. It is made of two main components: concepts and relationships.

- Concepts- Any major topics/findings/information from your article in circles or boxes
- Relationships- Concepts should be connected to one another with lines that are labeled with the relationship

There can be multiple relationships between concepts, showing the interconnectivity of ideas. See the example below:

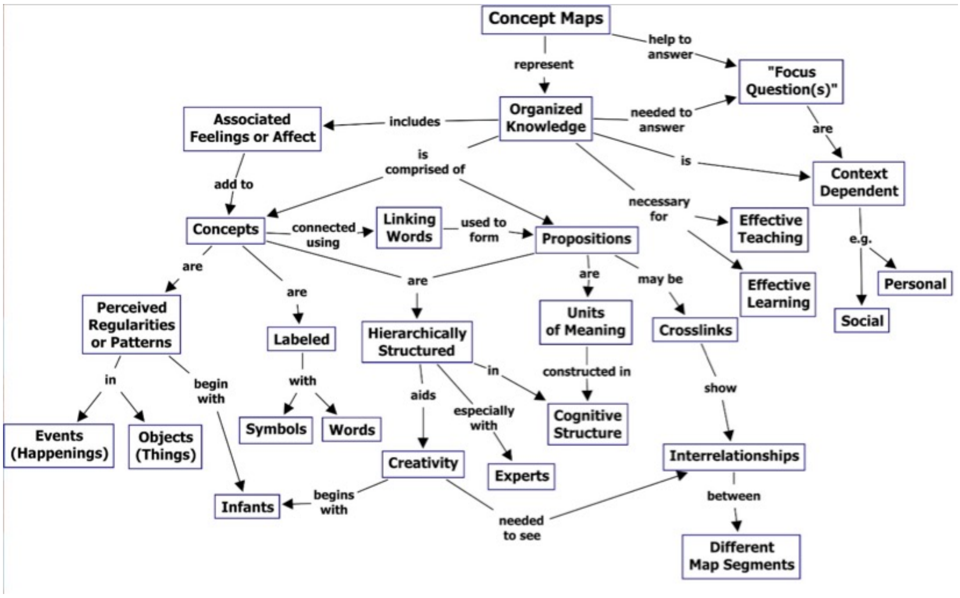

Electronic Concept Maps Resources:

MindMup-
https://www.mindmup.com

Lucid Chart-
https://www.lucidchart.com/pages/examples/concept-map-maker

Google Jamboard-
https://jamboard.google.com/

Google Draw-
https://docs.google.com/drawings/

Excalidraw-
https://excalidraw.com/

Student Handout

Piecing It All Together: Healthcare Disparities among People with Historically Excluded Identities

Name: Date:

Full Class Concept Map and Discussion:
As a class you will share your concept map and connect your ideas to a larger class concept map.
When it is your group’s turn share information that directly comes from your article:
- New concepts to add to the class's model from your article/concept map
- New relationships between concepts from your article/concept map
- Add any new concepts or lines between **existing** concepts for new relationships.

You can use the space below to record the class concept map or take notes if you need to.

Student Assessment

Piecing It All Together: Healthcare Disparities among People with Historically Excluded Identities

Name: Date:

1. Describe the activity. What was the issue you learned about?
2. What did this activity teach you about the relationship between biology and society?
3. How did the activity illustrate how human values influence science?
4. What are the different perspectives presented as part of the activity today?
5. How do your personal values or experiences relate to the themes presented in this activity, if at all? Has the activity impacted your personal values or views?
6. Did the activity make you think differently about the issue than before today's activity? How?
7. What are some causes of the healthcare disparities you learned about today?
8. What are some effects of the healthcare disparities you learned about today?
9. What are some solutions to the healthcare disparities you learned about today?
