# Supplementary material for: Teaching at the intersection of science and society: An activity on healthcare disparities
Source: Biol Methods Protoc. 2024 Jan 5;9(1):bpad041. doi: 10.1093/biomethods/bpad041 (PMC10833140; doi:10.1093/biomethods/bpad041)
Supplement: bpad041_Supplementary_Data [file bpad041_supplementary_data.zip › S4_DispHealth_slides.pptx]

## Slide 1
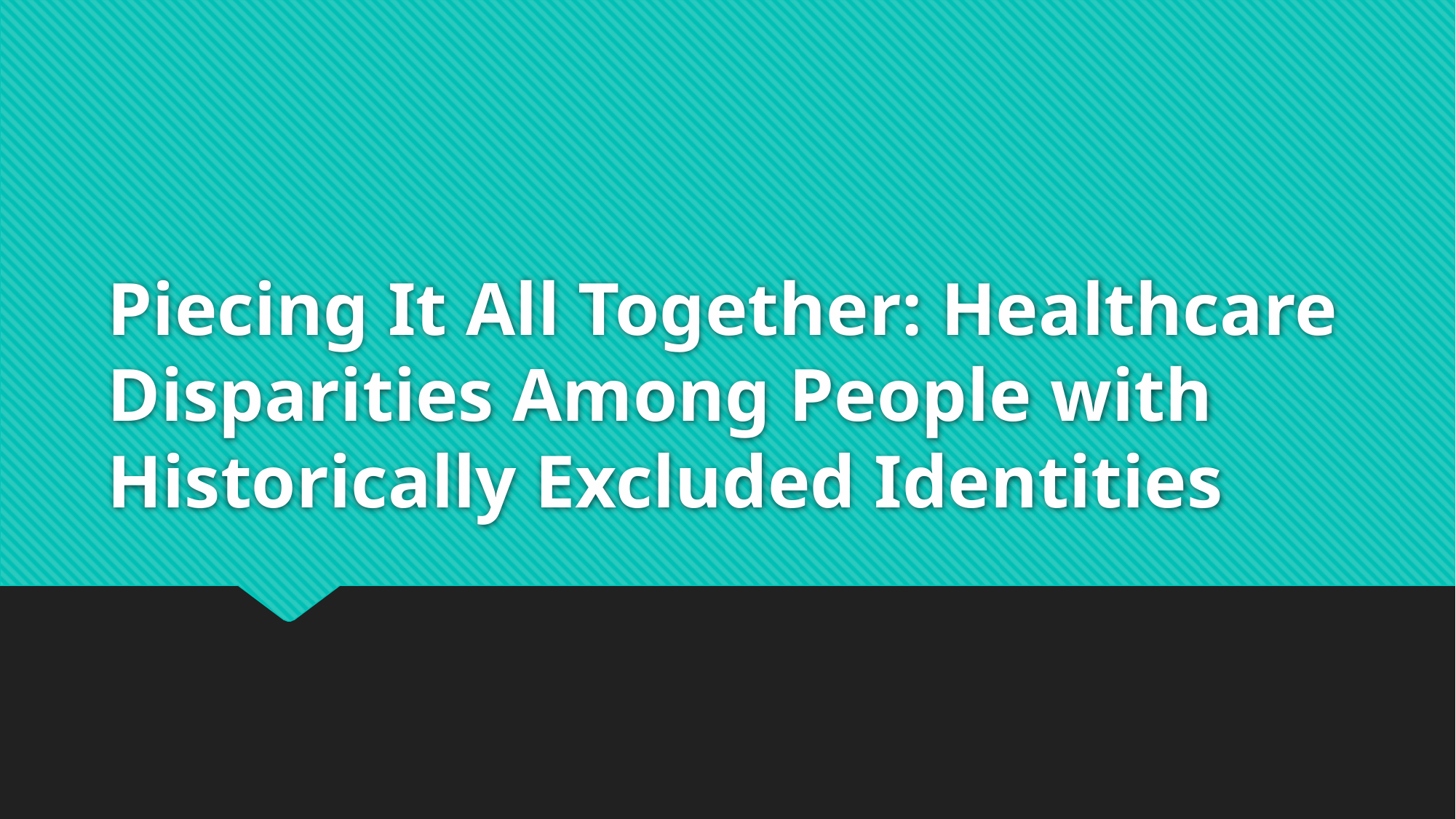

# Piecing It All Together: Healthcare Disparities Among People with Historically Excluded Identities

## Slide 2
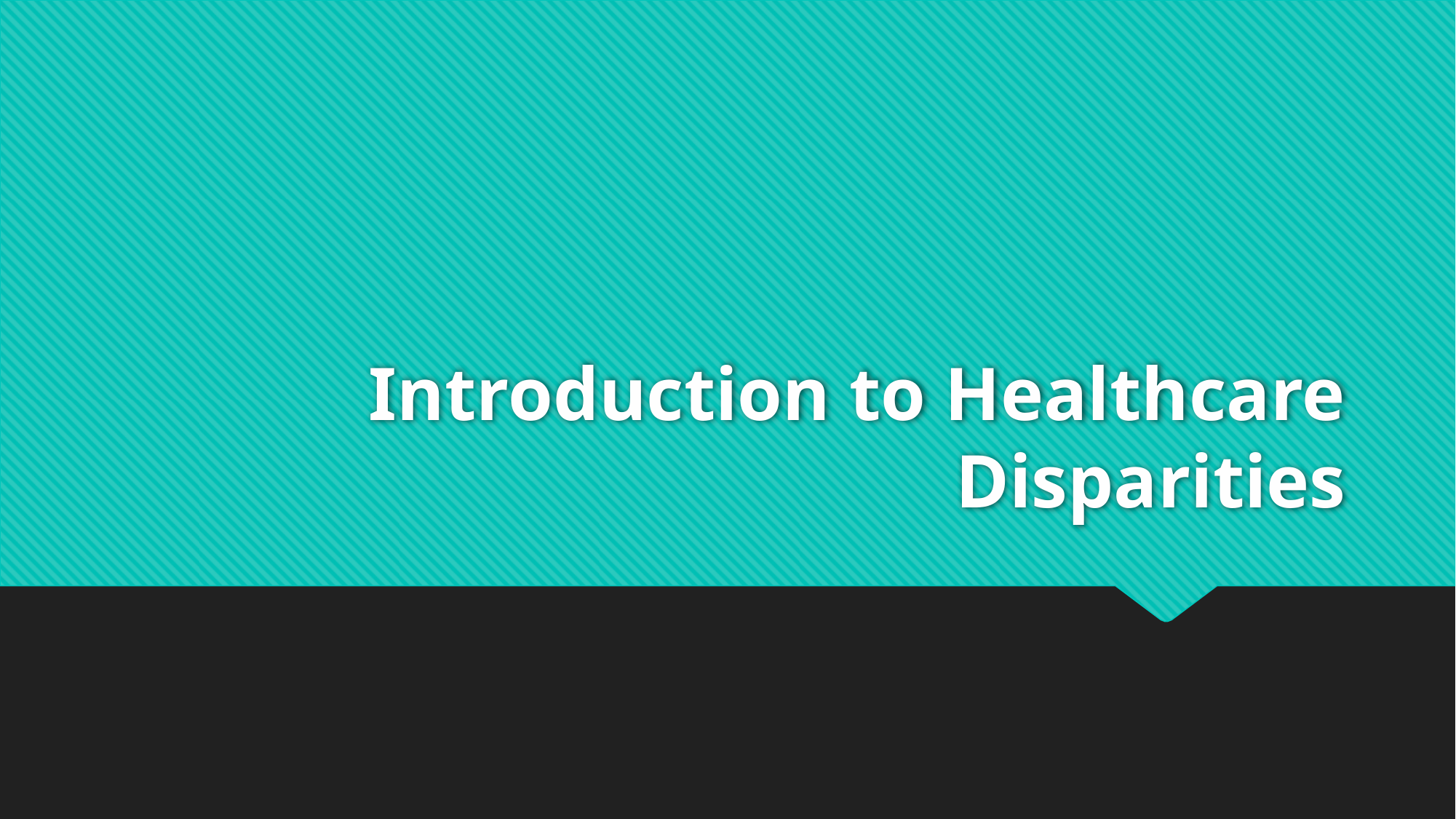

# Introduction to Healthcare Disparities

## Slide 3
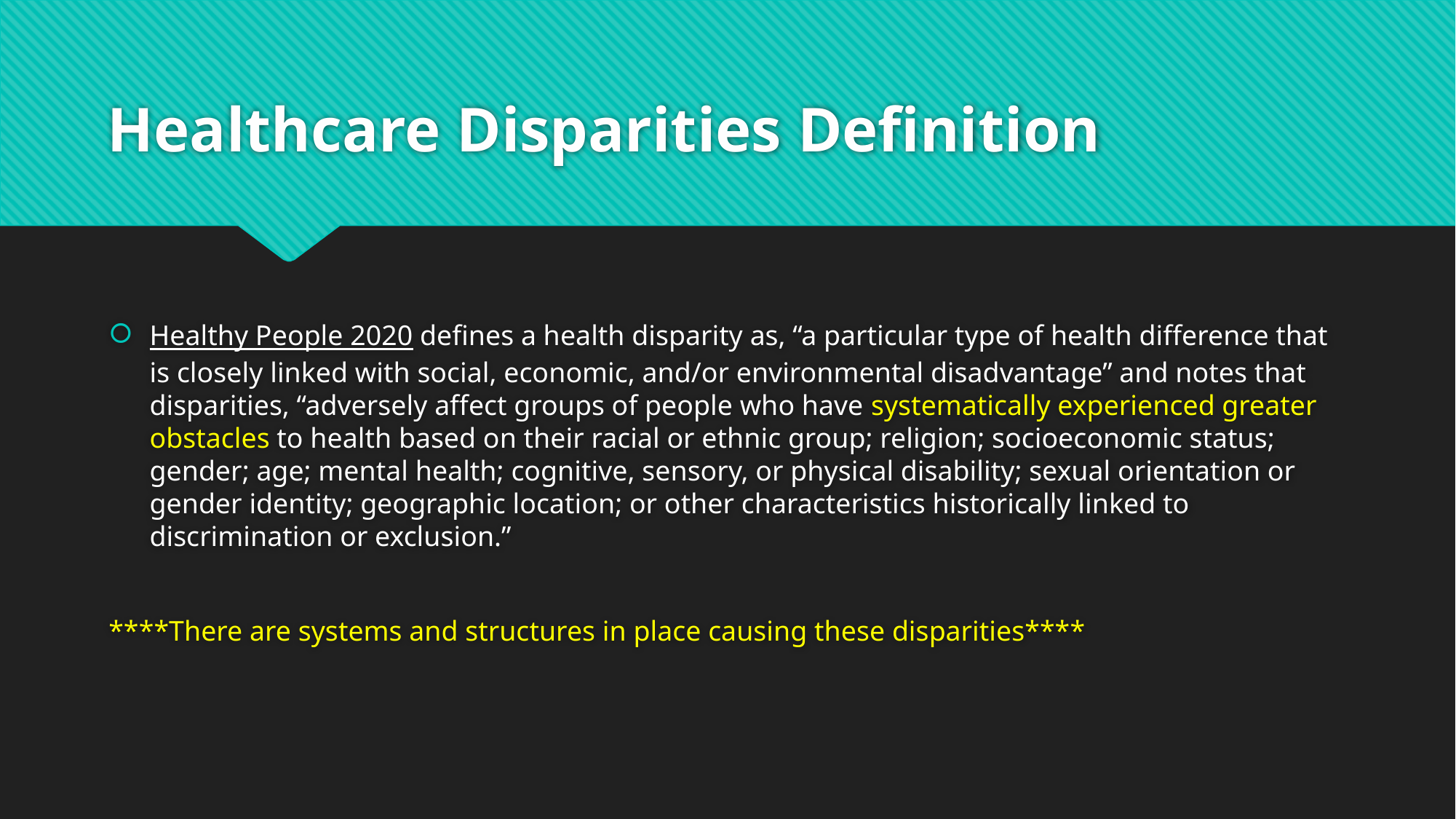

# Healthcare Disparities Definition
Healthy People 2020 defines a health disparity as, “a particular type of health difference that is closely linked with social, economic, and/or environmental disadvantage” and notes that disparities, “adversely affect groups of people who have systematically experienced greater obstacles to health based on their racial or ethnic group; religion; socioeconomic status; gender; age; mental health; cognitive, sensory, or physical disability; sexual orientation or gender identity; geographic location; or other characteristics historically linked to discrimination or exclusion.”
****There are systems and structures in place causing these disparities****

## Slide 4
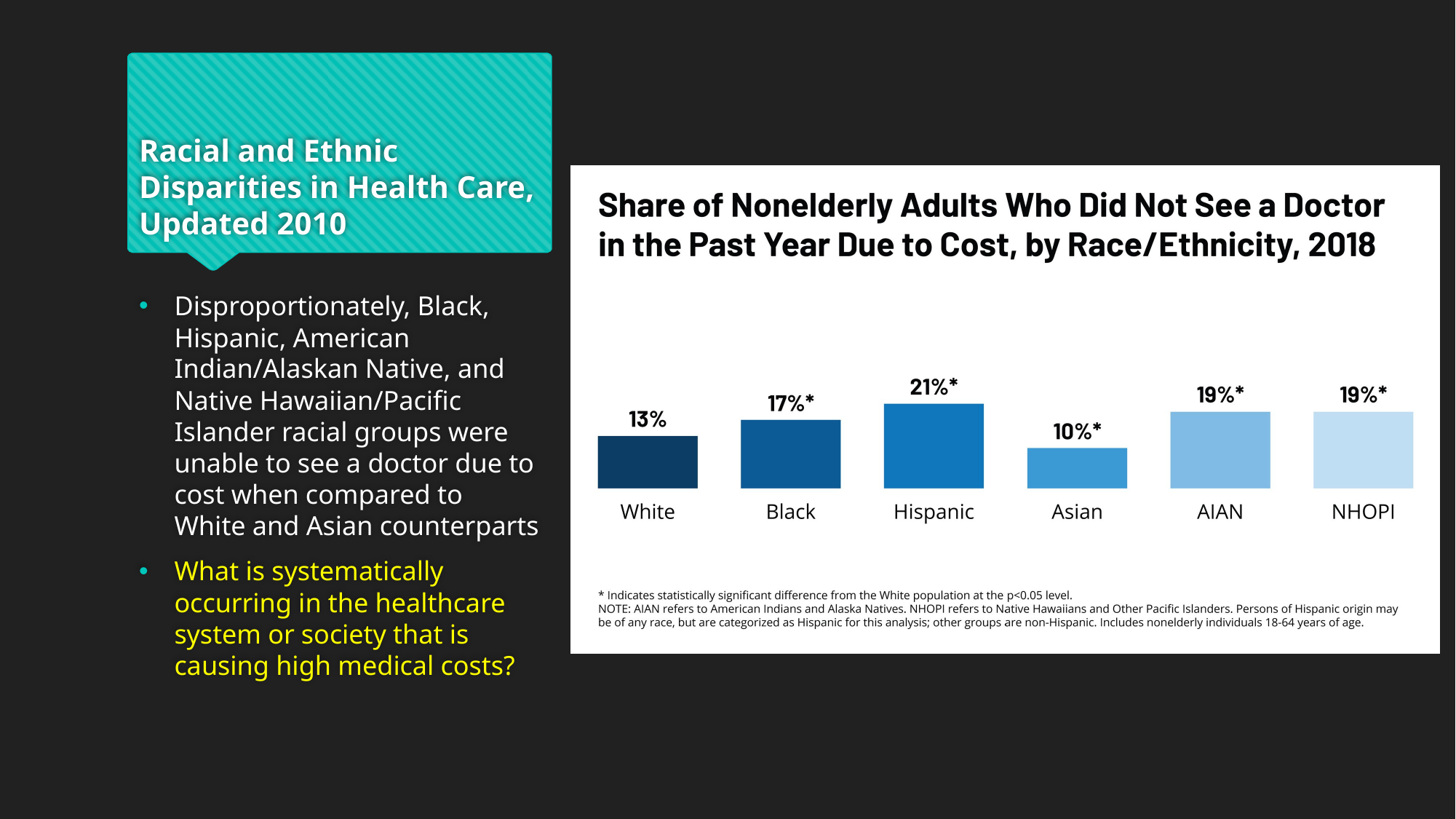

# Racial and Ethnic Disparities in Health Care, Updated 2010
Disproportionately, Black, Hispanic, American Indian/Alaskan Native, and Native Hawaiian/Pacific Islander racial groups were unable to see a doctor due to cost when compared to White and Asian counterparts
What is systematically occurring in the healthcare system or society that is causing high medical costs?

## Slide 5
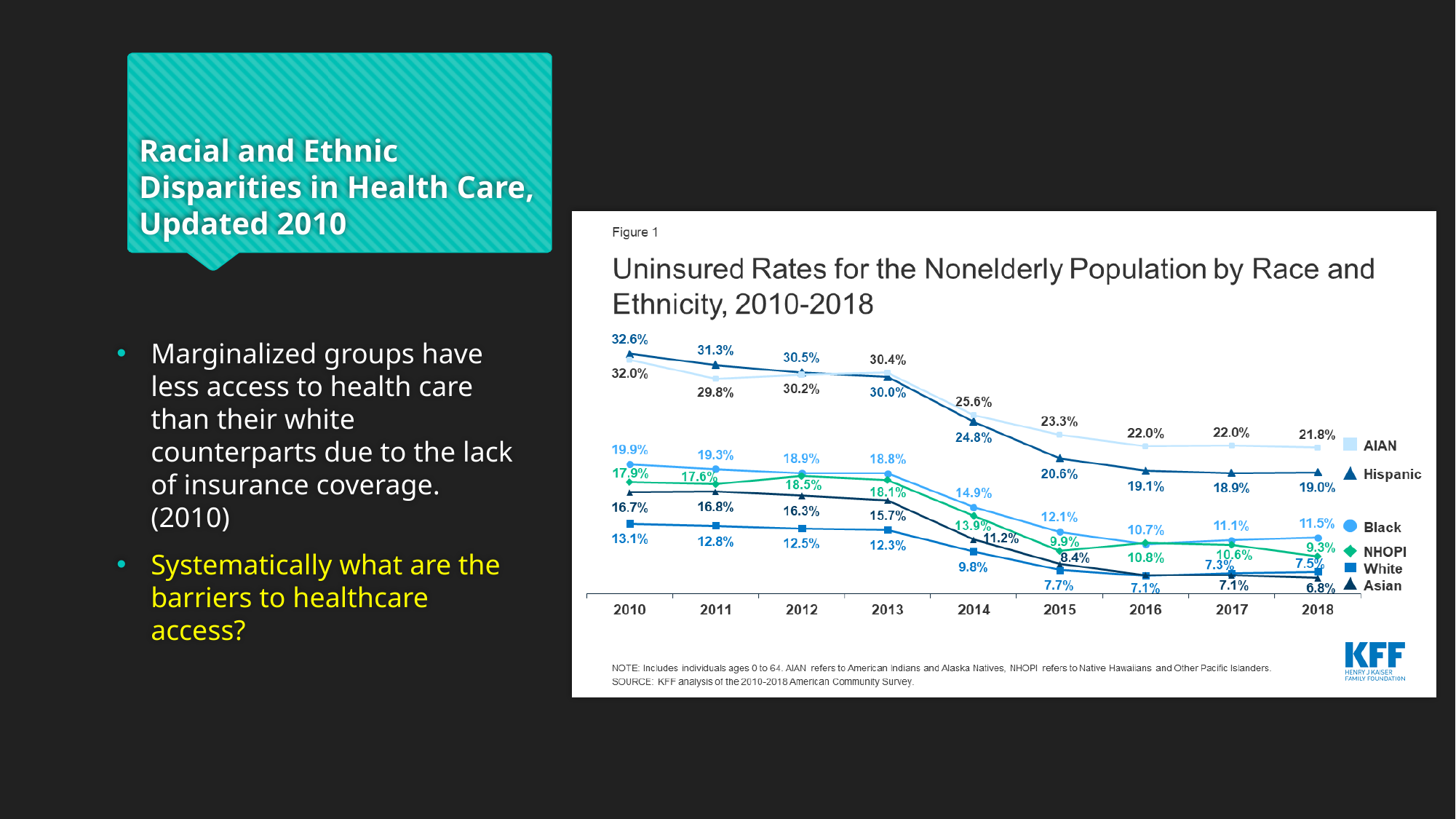

# Racial and Ethnic Disparities in Health Care, Updated 2010
Marginalized groups have less access to health care than their white counterparts due to the lack of insurance coverage. (2010)
Systematically what are the barriers to healthcare access?

## Slide 6
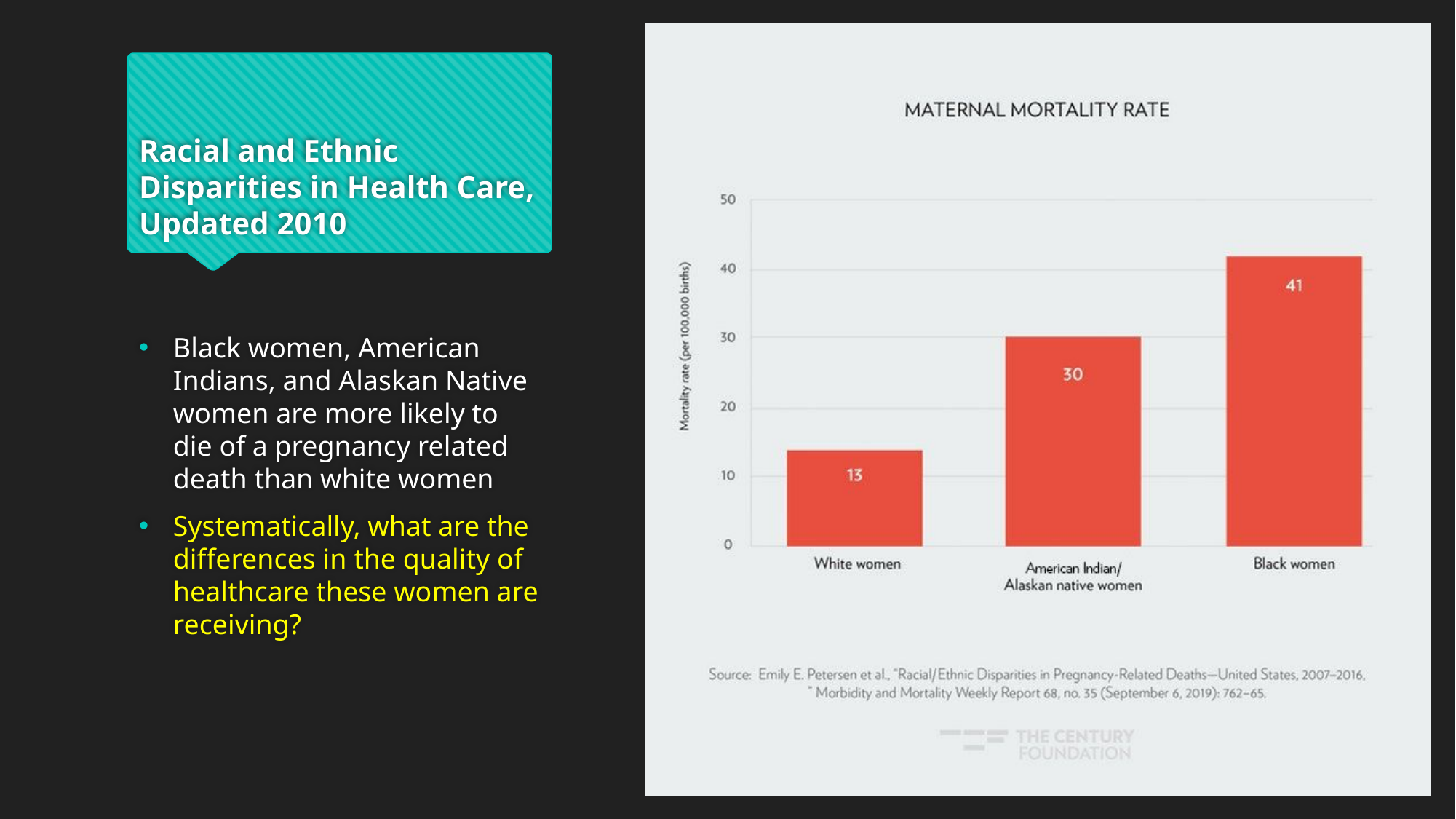

# Racial and Ethnic Disparities in Health Care, Updated 2010
Black women, American Indians, and Alaskan Native women are more likely to die of a pregnancy related death than white women
Systematically, what are the differences in the quality of healthcare these women are receiving?

## Slide 7
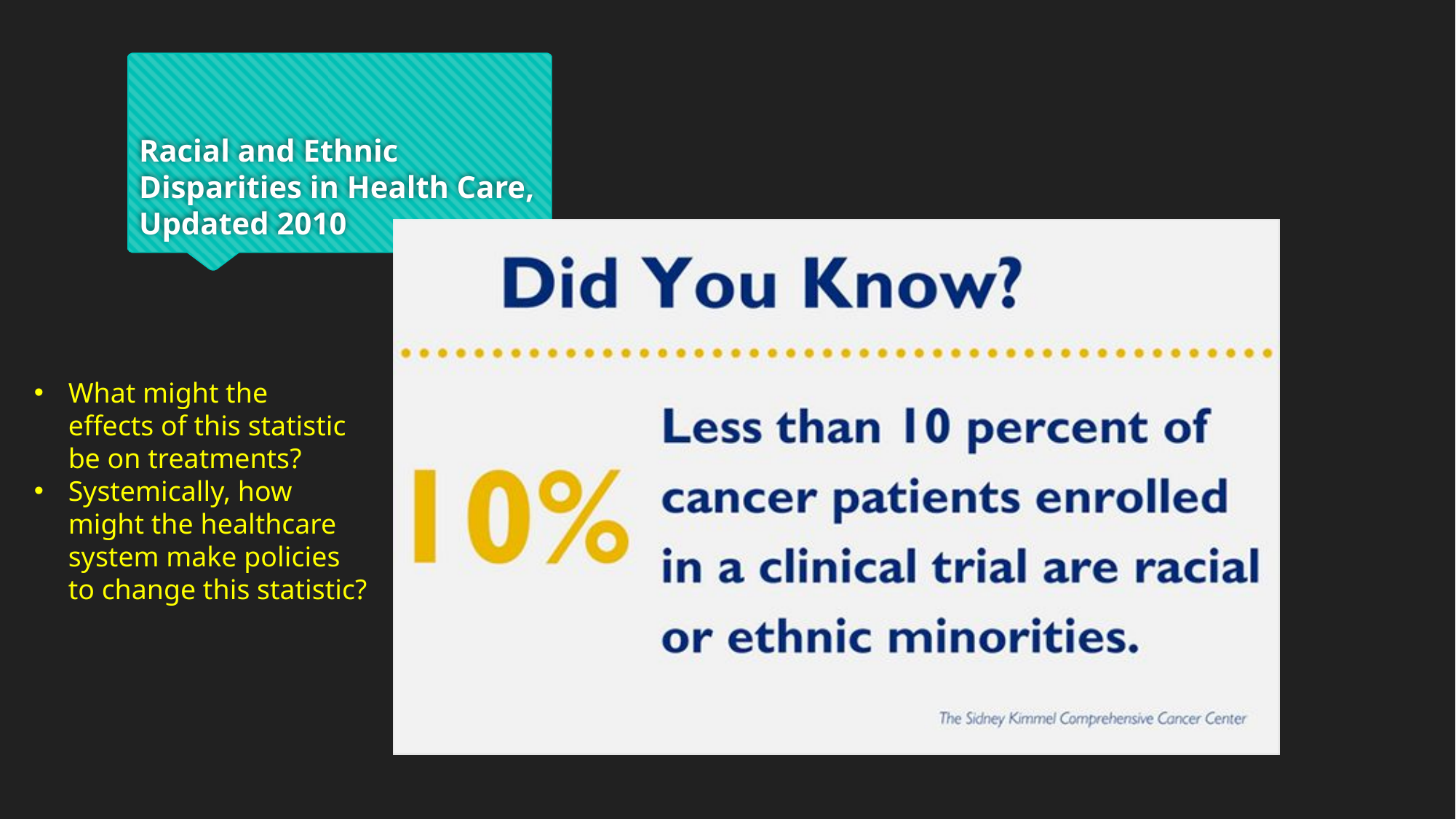

# Racial and Ethnic Disparities in Health Care, Updated 2010
What might the effects of this statistic be on treatments?
Systemically, how might the healthcare system make policies to change this statistic?

## Slide 8
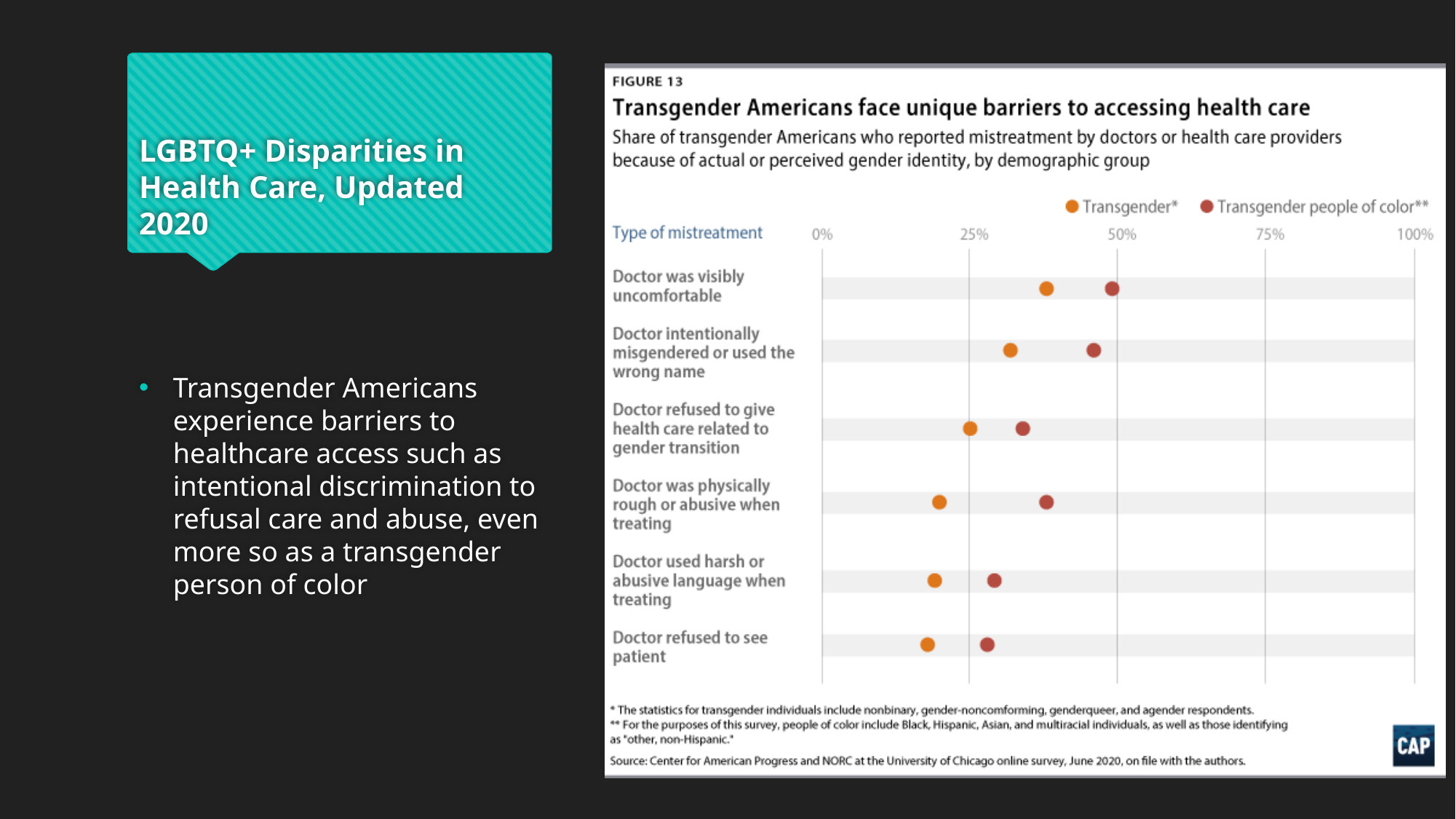

# LGBTQ+ Disparities in Health Care, Updated 2020
Transgender Americans experience barriers to healthcare access such as intentional discrimination to refusal care and abuse, even more so as a transgender person of color

## Slide 9
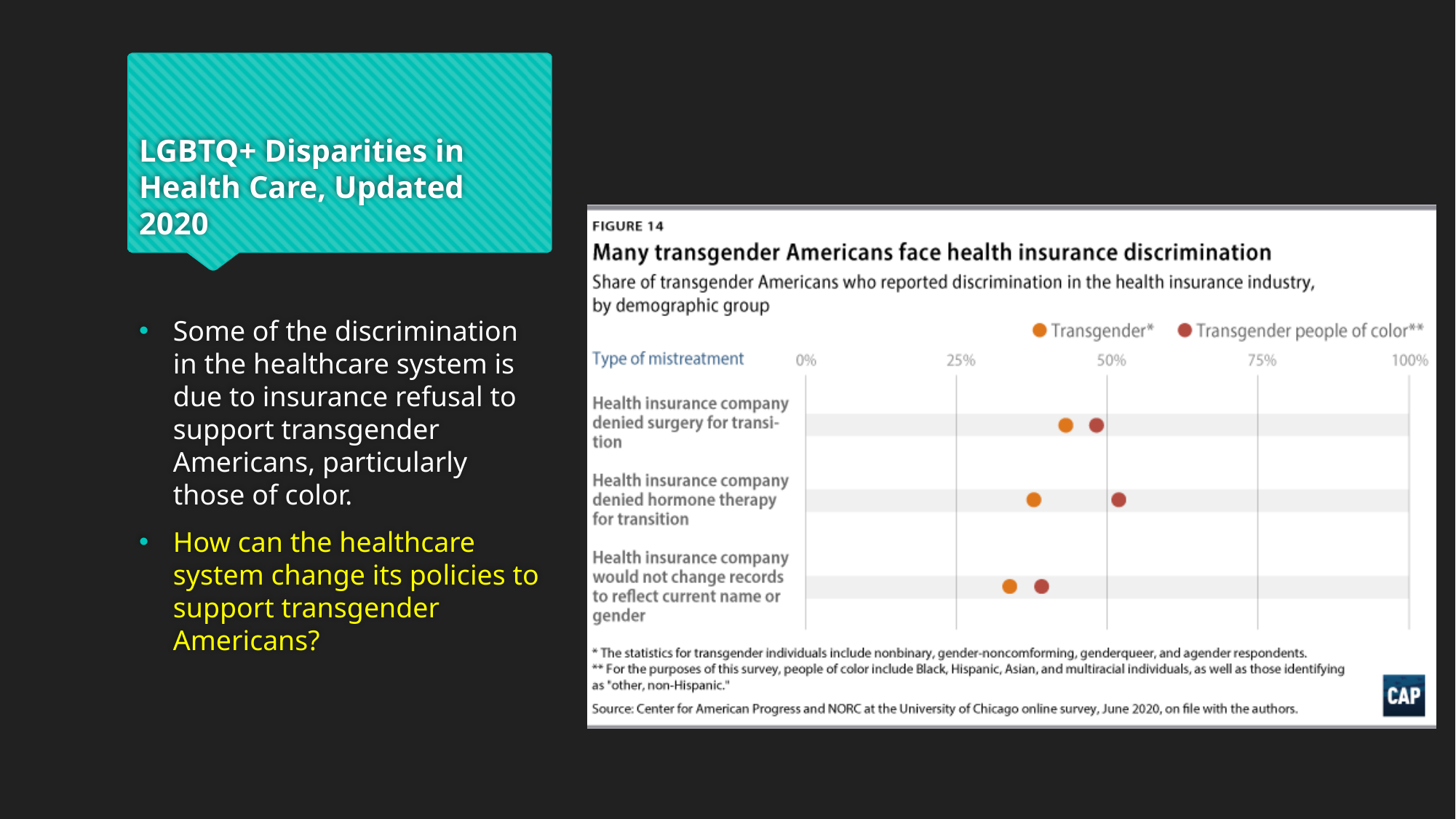

# LGBTQ+ Disparities in Health Care, Updated 2020
Some of the discrimination in the healthcare system is due to insurance refusal to support transgender Americans, particularly those of color.
How can the healthcare system change its policies to support transgender Americans?

## Slide 10
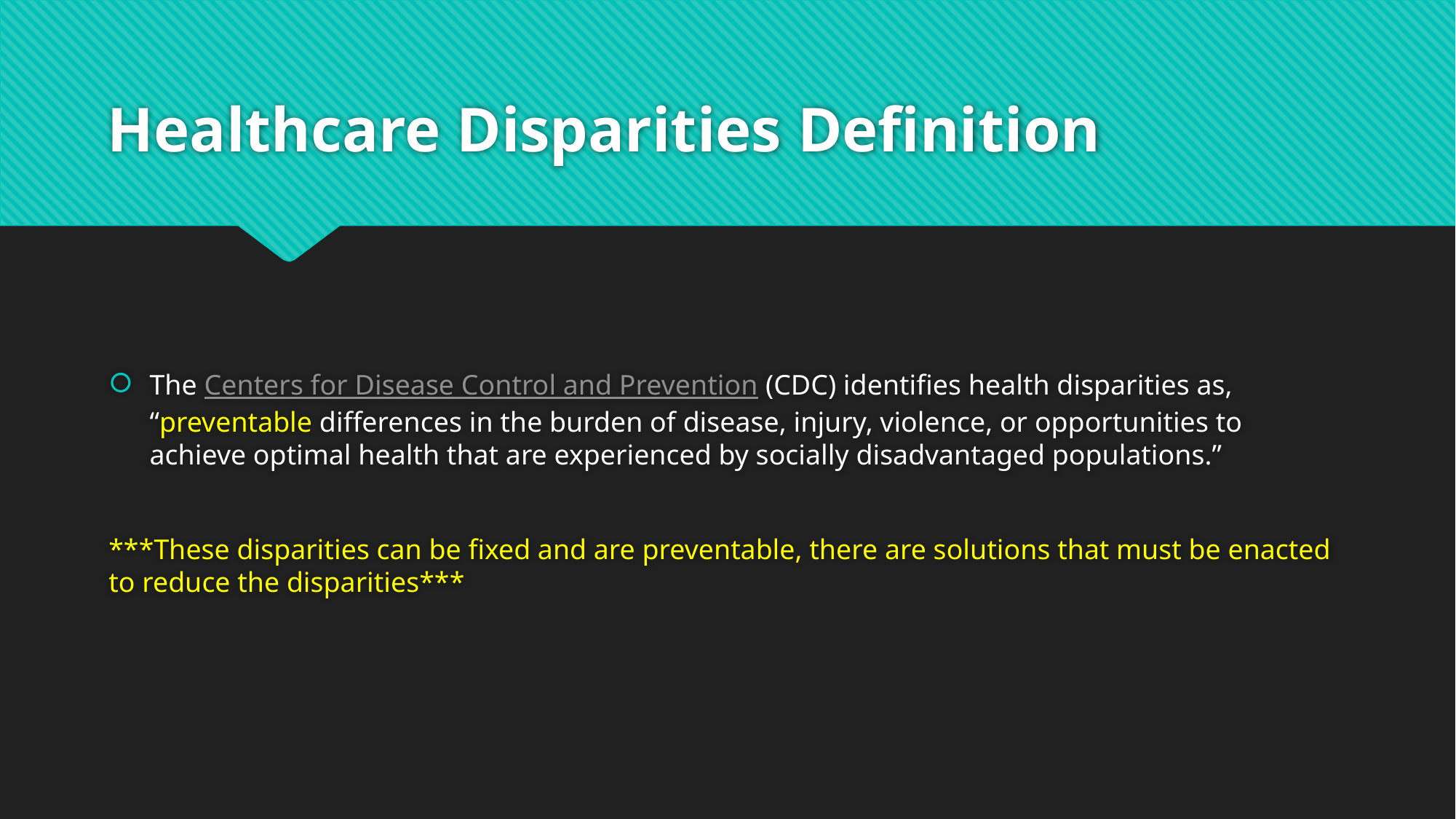

# Healthcare Disparities Definition
The Centers for Disease Control and Prevention (CDC) identifies health disparities as, “preventable differences in the burden of disease, injury, violence, or opportunities to achieve optimal health that are experienced by socially disadvantaged populations.”
***These disparities can be fixed and are preventable, there are solutions that must be enacted to reduce the disparities***

## Slide 11
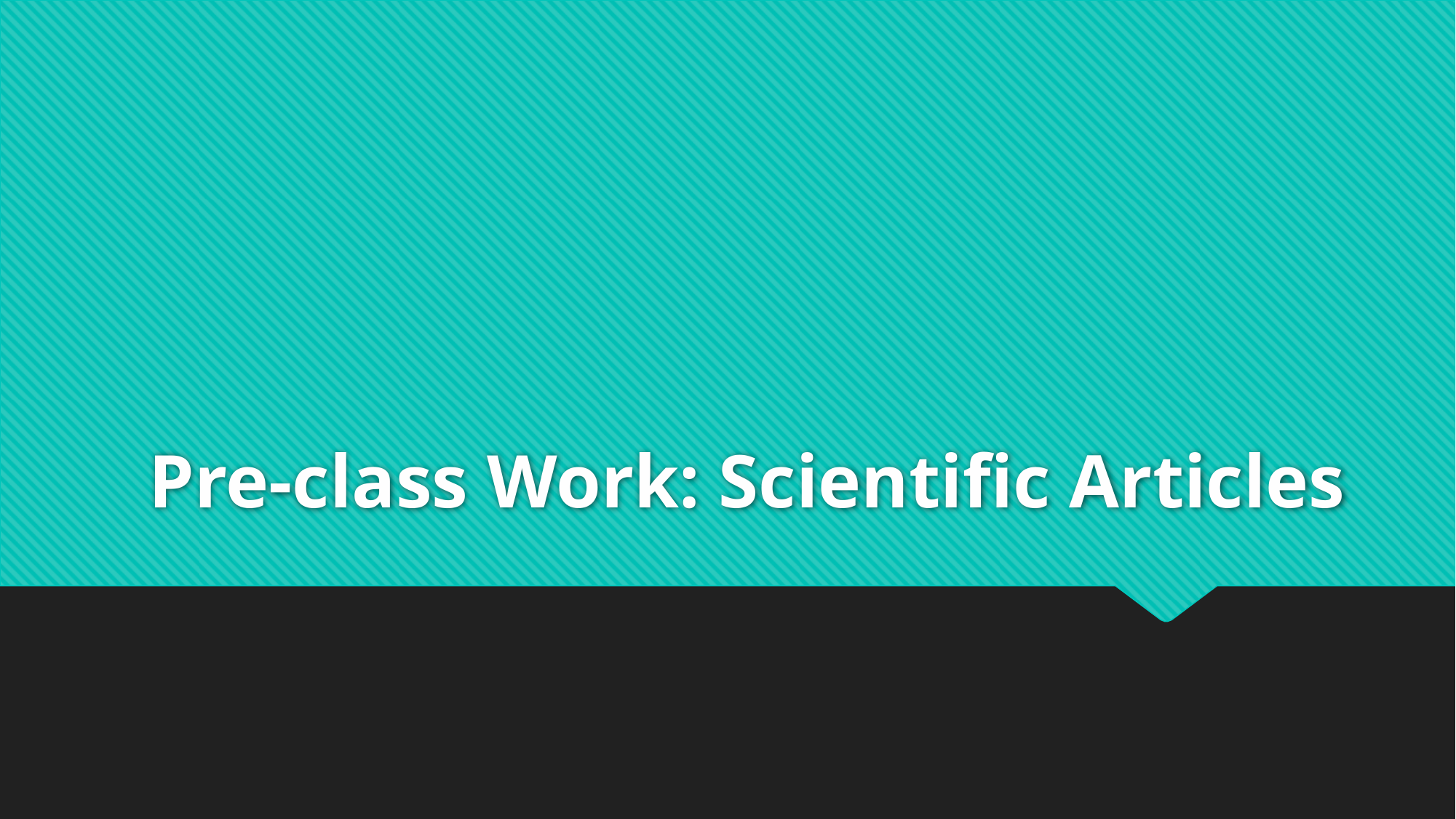

# Pre-class Work: Scientific Articles

## Slide 12
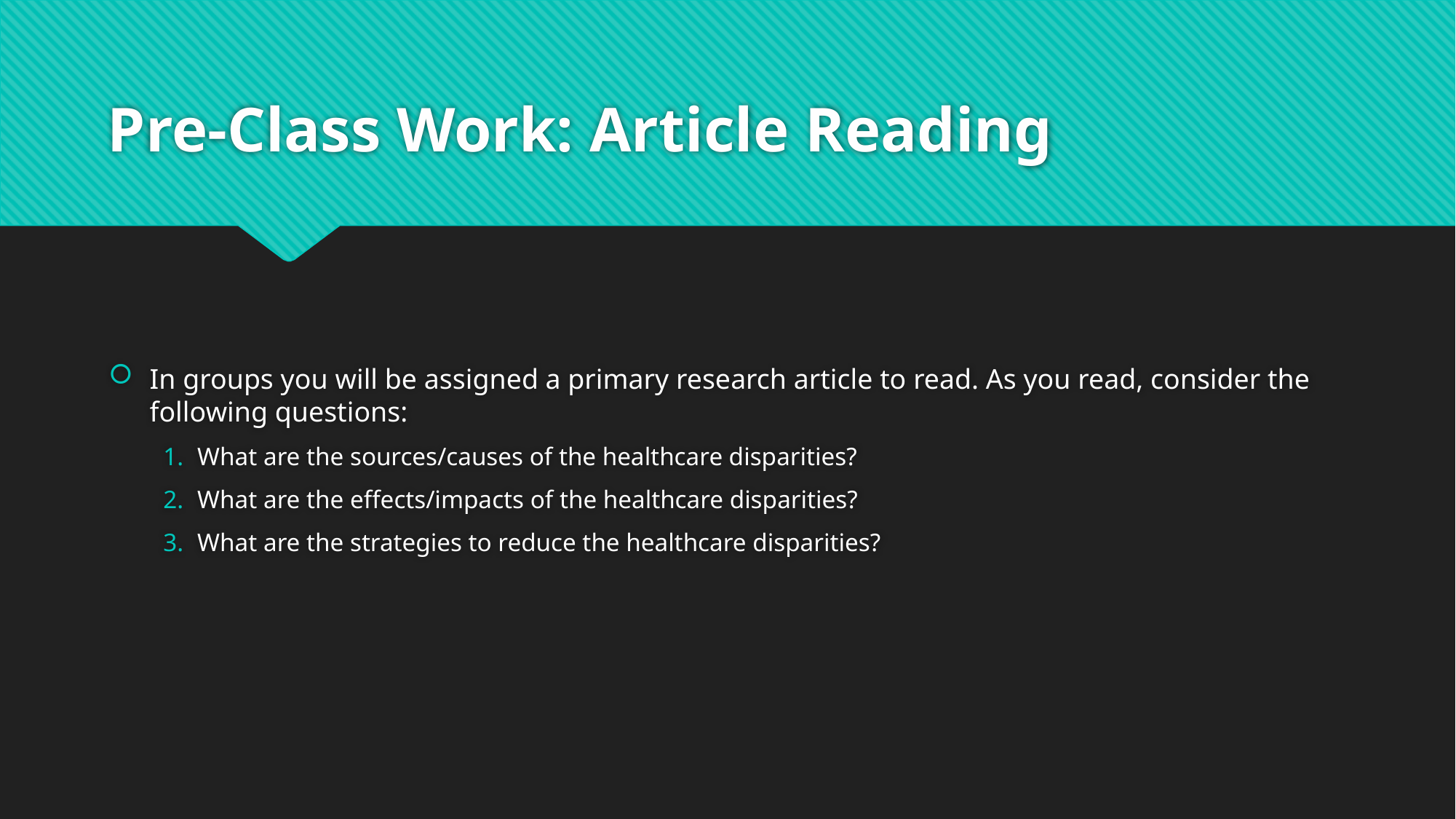

# Pre-Class Work: Article Reading
In groups you will be assigned a primary research article to read. As you read, consider the following questions:
What are the sources/causes of the healthcare disparities?
What are the effects/impacts of the healthcare disparities?
What are the strategies to reduce the healthcare disparities?

## Slide 13
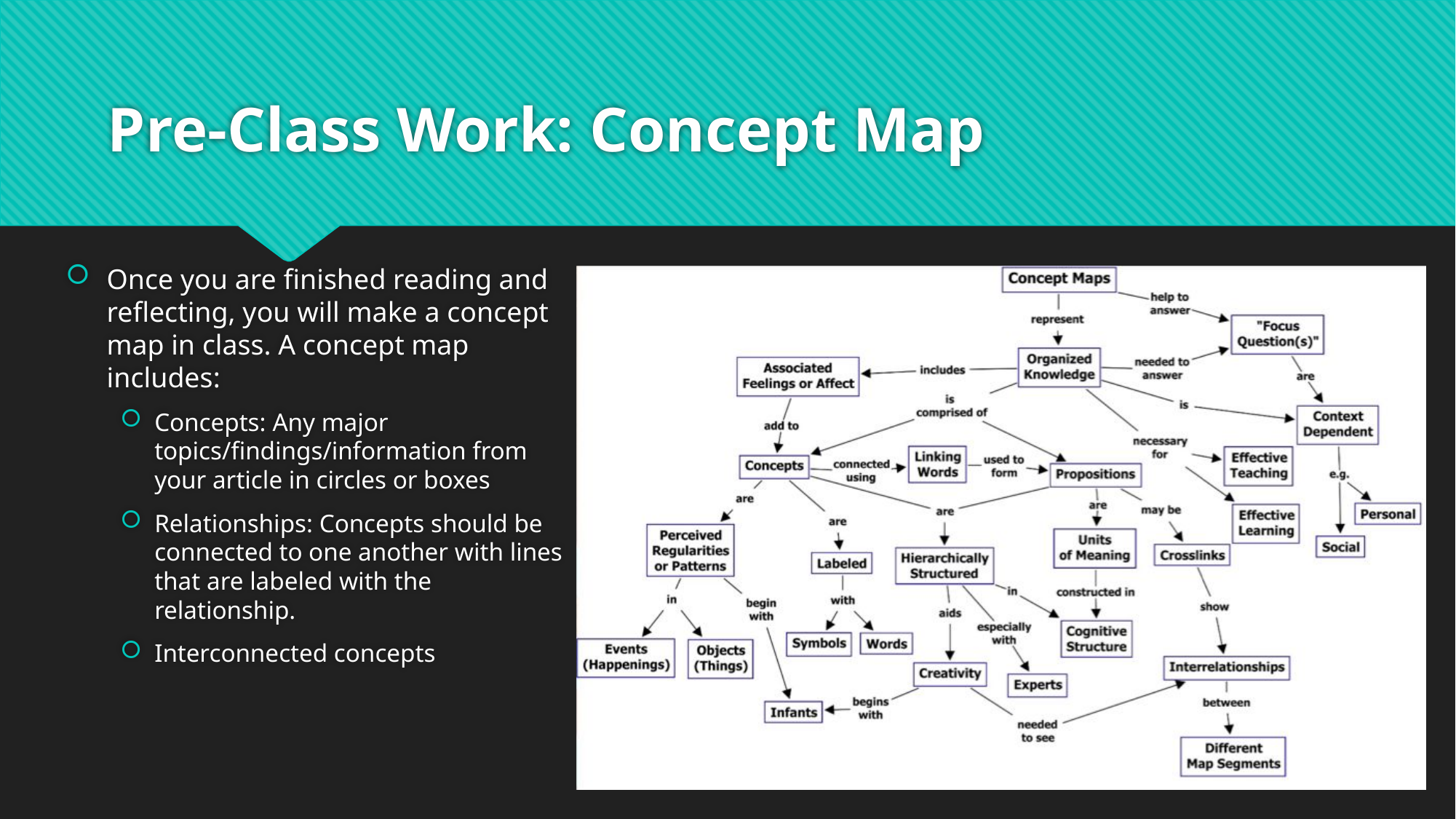

# Pre-Class Work: Concept Map
Once you are finished reading and reflecting, you will make a concept map in class. A concept map includes:
Concepts: Any major topics/findings/information from your article in circles or boxes
Relationships: Concepts should be connected to one another with lines that are labeled with the relationship.
Interconnected concepts

## Slide 14
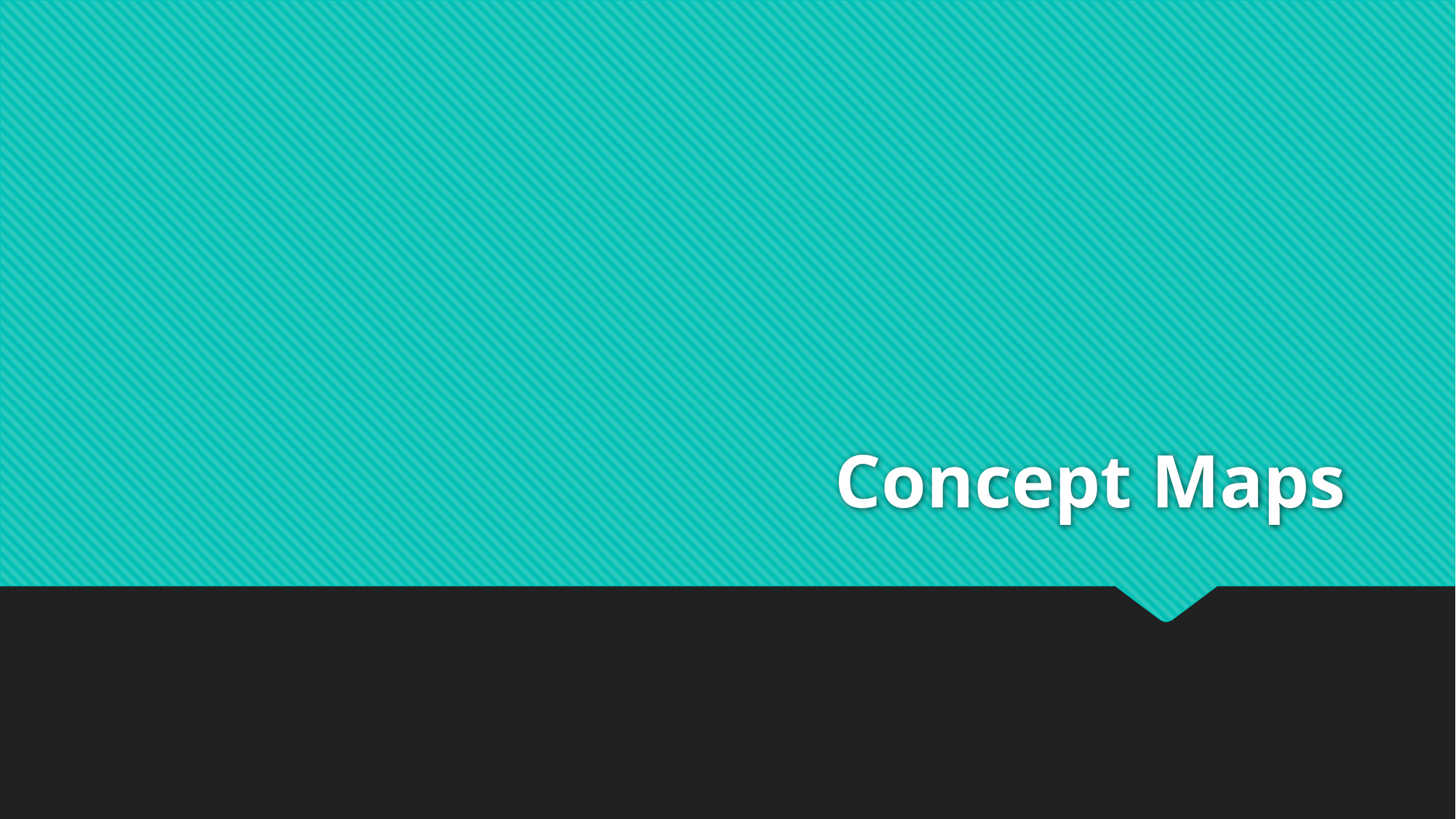

# Concept Maps

## Slide 15
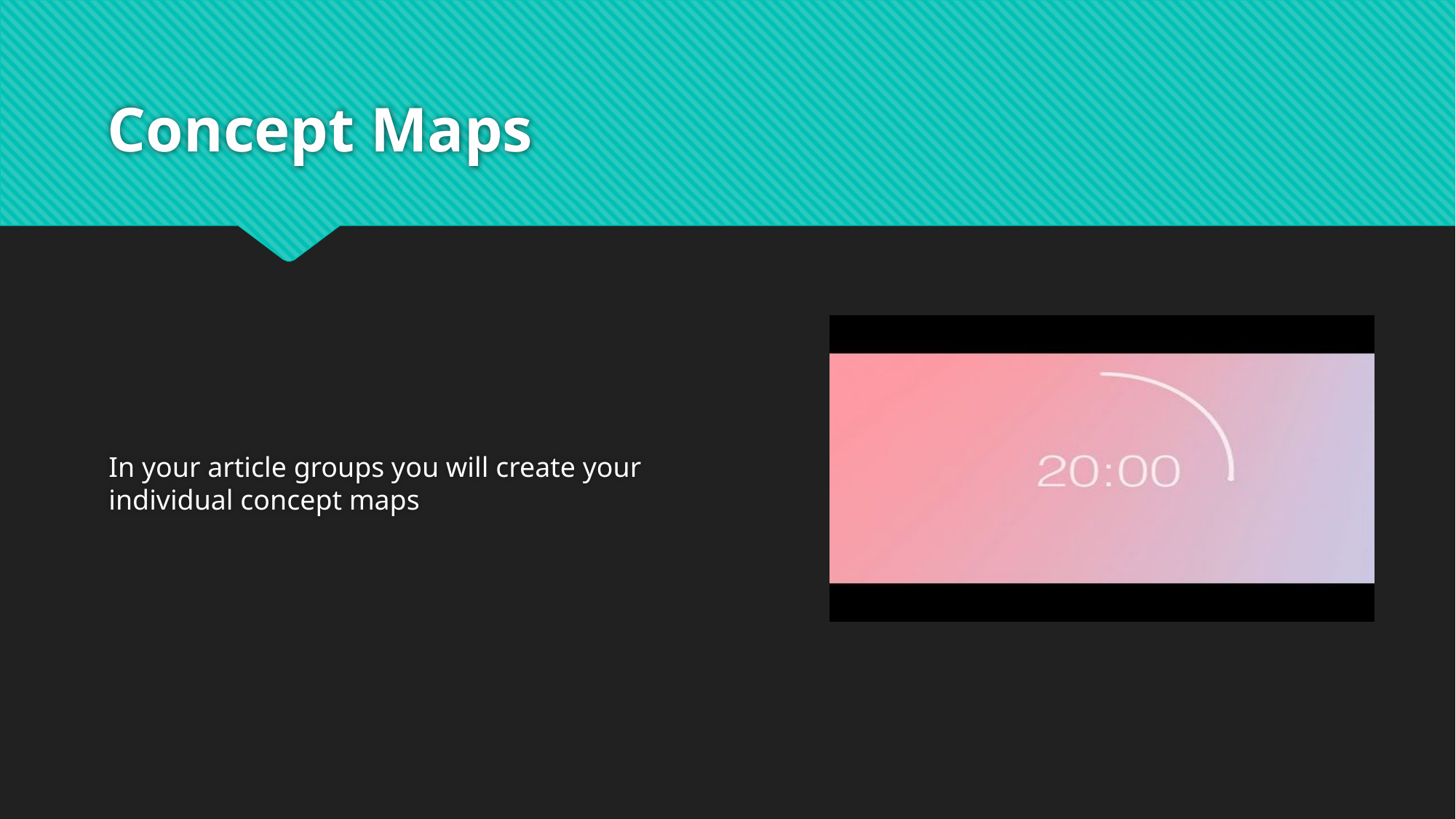

# Concept Maps
In your article groups you will create your individual concept maps

## Slide 16
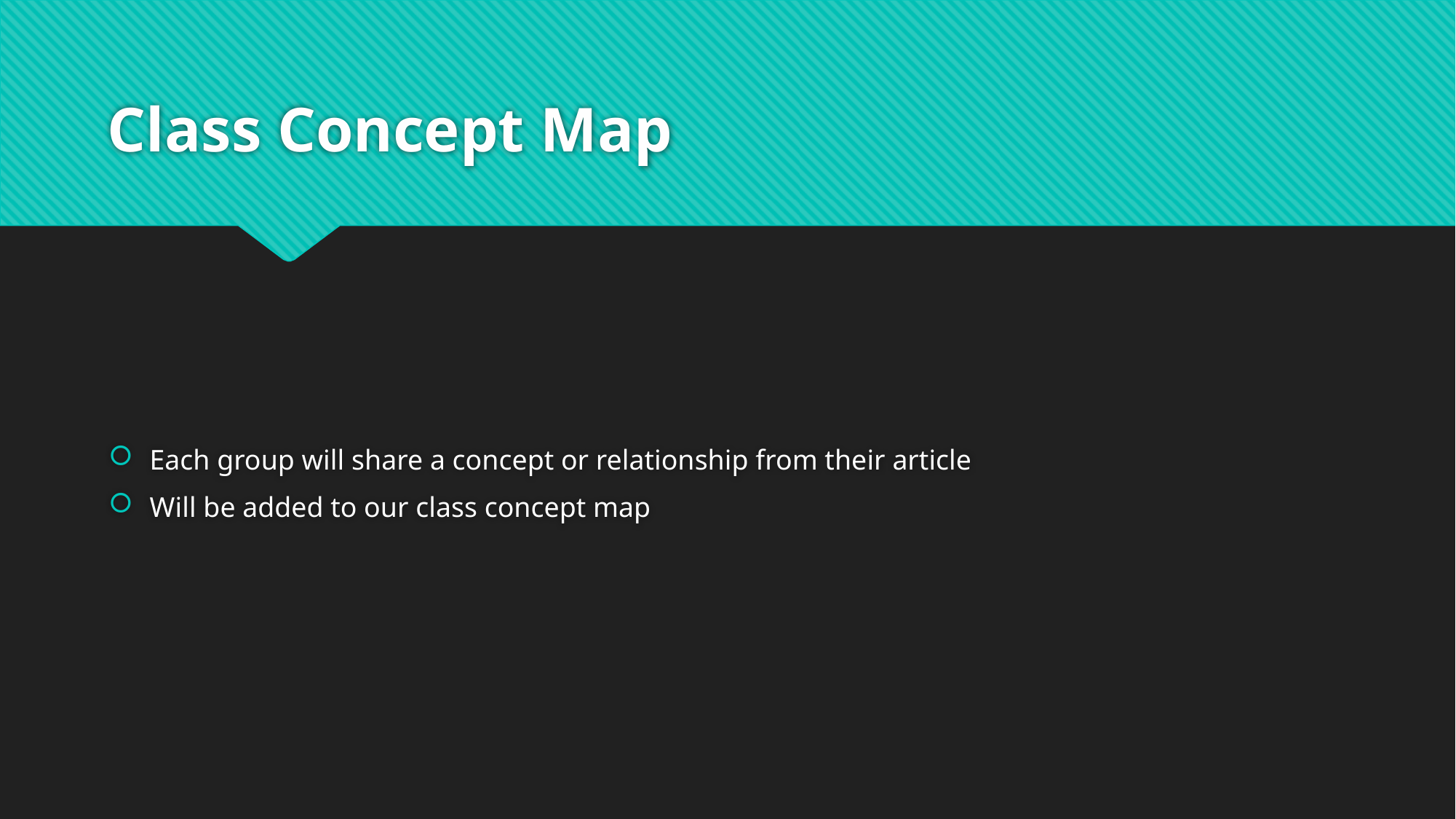

# Class Concept Map
Each group will share a concept or relationship from their article
Will be added to our class concept map

## Slide 17
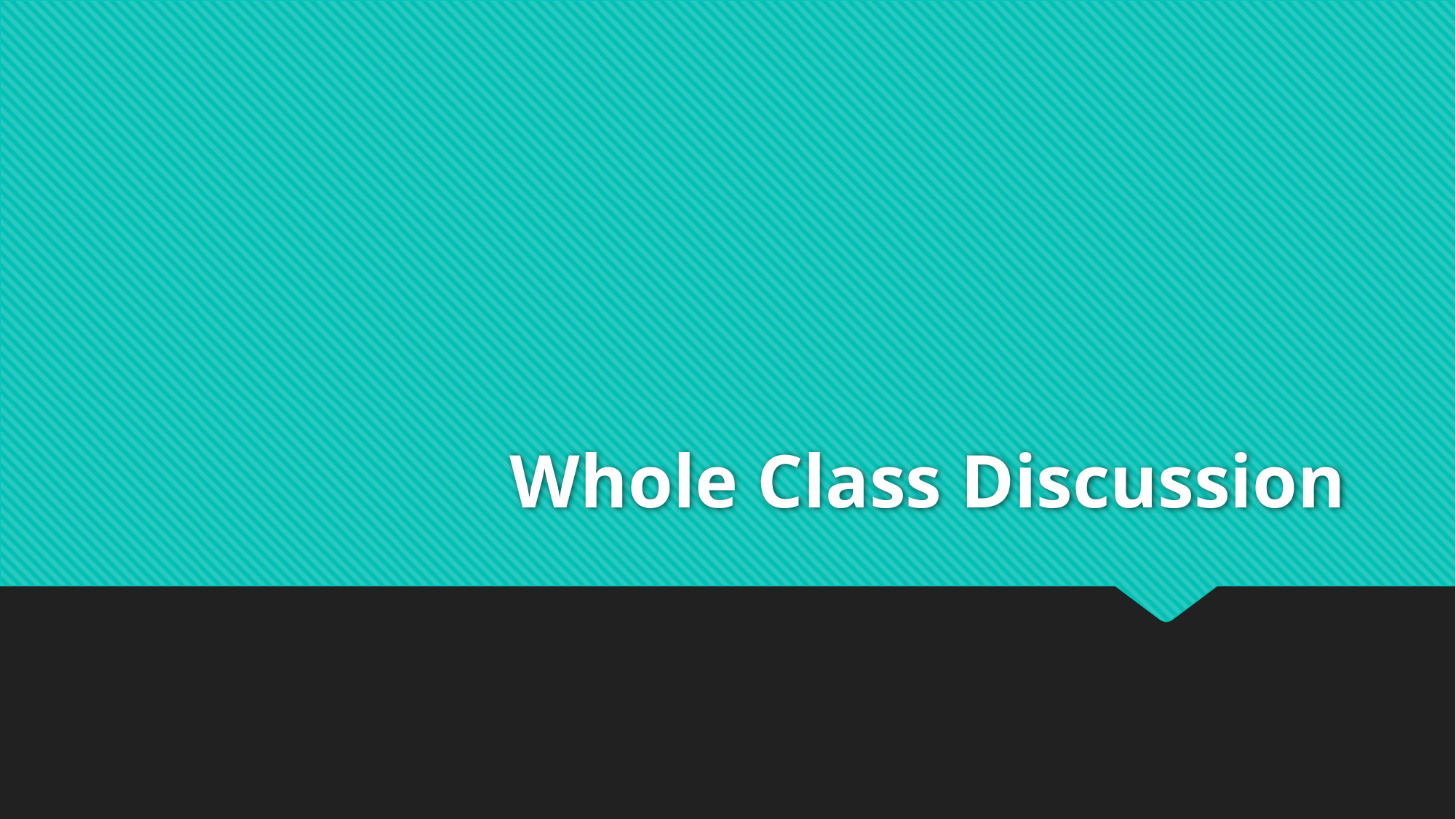

# Whole Class Discussion

## Slide 18
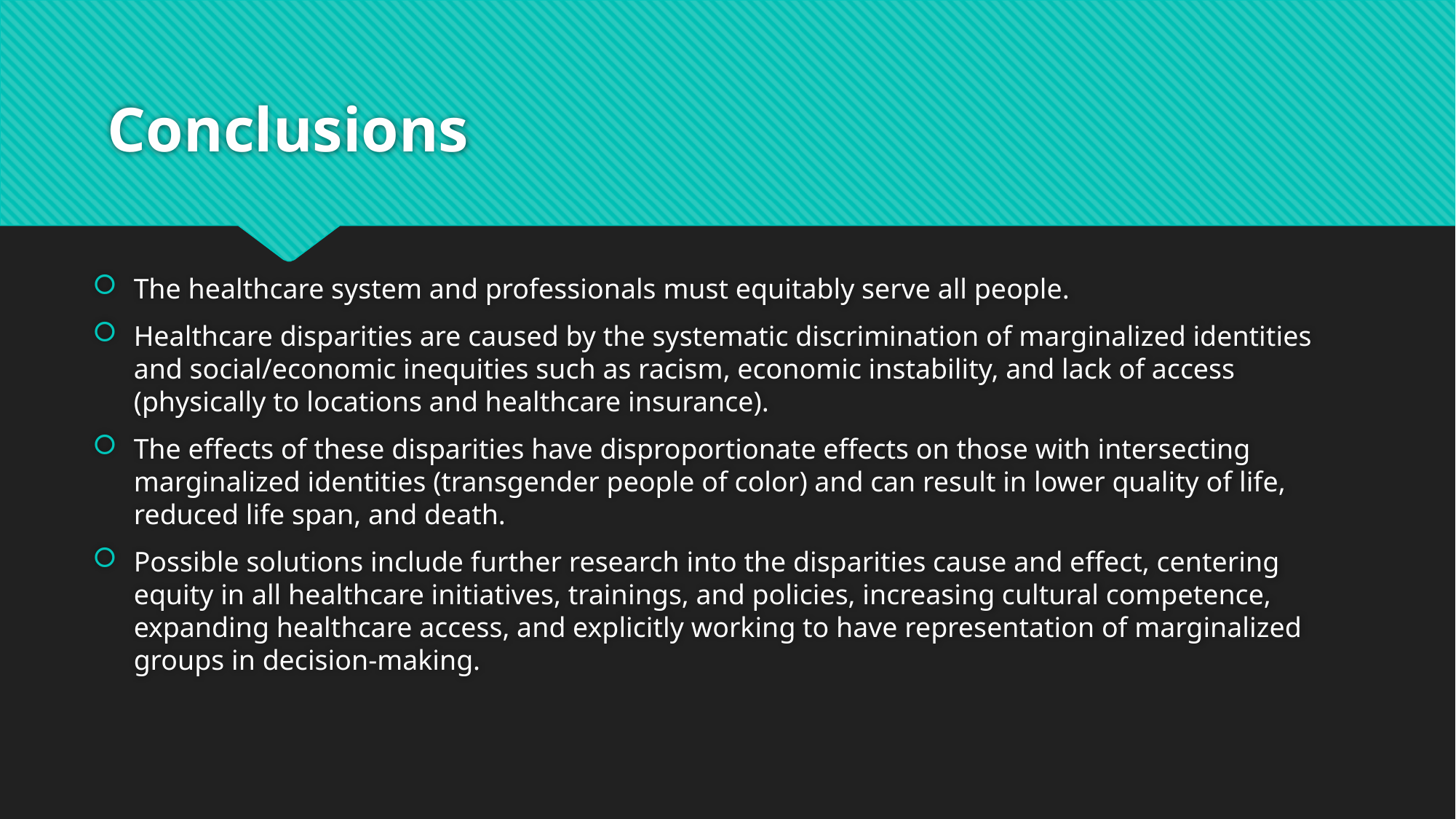

# Conclusions
The healthcare system and professionals must equitably serve all people.
Healthcare disparities are caused by the systematic discrimination of marginalized identities and social/economic inequities such as racism, economic instability, and lack of access (physically to locations and healthcare insurance).
The effects of these disparities have disproportionate effects on those with intersecting marginalized identities (transgender people of color) and can result in lower quality of life, reduced life span, and death.
Possible solutions include further research into the disparities cause and effect, centering equity in all healthcare initiatives, trainings, and policies, increasing cultural competence, expanding healthcare access, and explicitly working to have representation of marginalized groups in decision-making.

## Slide 19
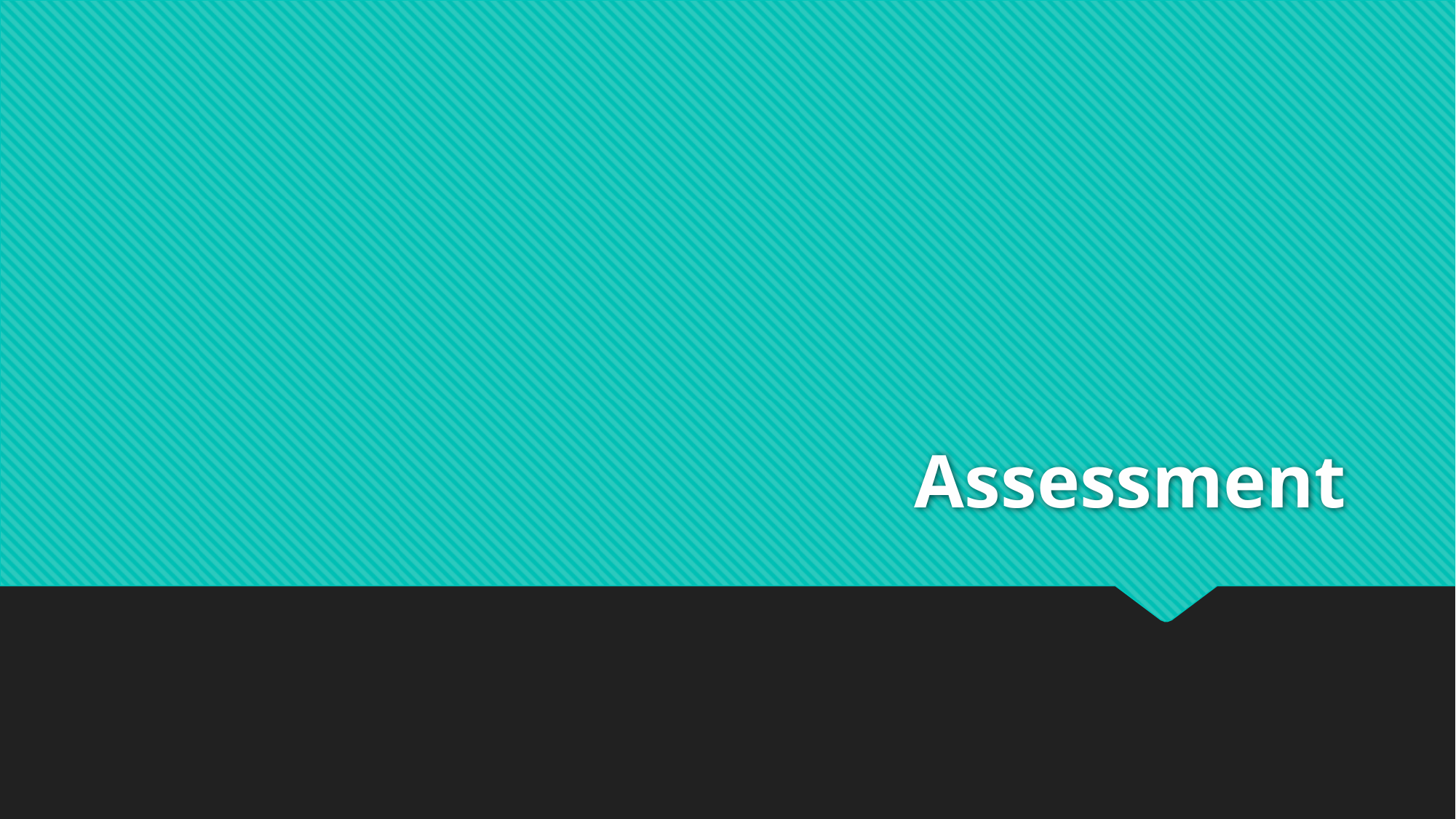

# Assessment
