# Supplementary material for: Teaching at the intersection of science and society: An activity on healthcare disparities
Source: Biol Methods Protoc. 2024 Jan 5;9(1):bpad041. doi: 10.1093/biomethods/bpad041 (PMC10833140; doi:10.1093/biomethods/bpad041)
Supplement: bpad041_Supplementary_Data [file bpad041_supplementary_data.zip › S1_Supplement_alternate timeline.docx]

**Supplementary Materials S1**

**Alternate Timeline**

In a 50-minute class period, the activity will need to be split over two days. **Supplemental Table 1** shows a proposed timeline for implementing this in in a 50 minute class period. The first day requires the introductory lecture (~15 minutes) which could also include a detailed introduction to concept mapping if students are unfamiliar. If you do not introduce concept mapping in the first lecture, you will still have ~30-35 minutes of class period for your normal course material. Students will read their assigned articles for homework and review materials on concept mapping. On day two, students will break into their groups to build their individual concept maps and discuss their assigned article (~20 minutes). You will then dissolve the groups, and bring the students back together to build a full-class concept map covering all of the assigned articles (~20 minutes). End the class with a wrap-up discussion, and assign the assessment to be completed for homework.

**Supplemental Table 1.** Alternate timeline of course activities.

| **Activity** | **Description** | **Est Time** | **Notes** |
| --- | --- | --- | --- |
| **Class Session Part I** | | | |
| Introductory Lecture | The instructor reviews healthcare inequalities and connects them to prior knowledge throughout. The instructor introduces concept mapping (if new for this class). | >15 mins | *PowerPoint*  *included* |
| **Student Preparation for Class Part II** | | | |
| Students read selected articles | Student in groups are assigned an article to read. | >2 hours |  |
| Concept Map Introduction | Students will watch a video and read instructions on concept mapping. |  |  |
| **Class Session Part II** | | | |
| Collaborative Work: Concept Mapping | Students break into groups to discuss their assigned scientific article, and build their individual concept maps. If students are unfamiliar with concept mapping, instructor can provide guidance as the students work in groups. | >20 mins | *Electronically*  *or with*  *paper/pencil* |
| Full Class Concept-Map | Dissolve groups; The class will work together to discuss their scientific articles and build a concept map combining ALL of the scientific articles. | >20 mins | *Electronically*  *or with*  *paper/pencil* |
| Discussion & Wrap-Up | End the class with a discussion of the central themes/ideas that surround the idea of healthcare inequalities. | >5 mins |  |
| Assessment | Students complete the post-activity assessment for homework. | ~15 mins | *Included in student hand-out* |
